# Monkeypox in 2022: A new threat in developing

**DOI:** 10.1016/j.amsu.2022.103975

**Published:** 2022-06-07

**Authors:** Mansoor Ahmed, Haseena Naseer, Mateen Arshad, Afnan Ahmad

**Affiliations:** a Holy Family Hospital, Rawalpindi Medical University, New Teaching Block, RMU, Satellite Town, Rawalpindi, Punjab, 46000 , Pakistan; b Fauji Foundation Hospital, Foundation University Medical College, Defence Ave, DHA Phase-I Islamabad, Rawalpindi, Punjab, 44000 , Pakistan

**Keywords:** Monkey pox, Viral Zoonose, Zoonotic diseases, Viral

## Abstract

Monkeypox that started spreading from central and western Africa has recently been detected in various countries including Spain, Canada, Australia, and UAE. Though it has a very low incidence rate, it is still a significant threat that must be dealt with as quickly as possible and the probability of it spreading to many other countries is still very high. It is caused the by Monkeypox virus that belongs to the genus Orthopox that also include smallpox virus, which has quite similar clinical presentation as that of Monkeypox. With fading immunity against Smallpox, it is important to counter this threat with newly developed vaccines.

Monkeypox virus belongs to the genus Orthopox of the family Poxviridae. Orthopox also includes smallpox virus, cowpox virus, rabbitpox virus. These are transmitted by contact with infected animals and are usually seen in occupational settings. Monkeypox is closely related to smallpox, and the vaccine against smallpox is reported to be protective against Monkeypox. Monkeypox was first discovered in monkeys. The first case of Monkeypox pox was reported in the democratic republic of Congo in 1970 and has spread to various countries inside and outside Africa since then, but is mainly confined to central and Western African countries. There are two genetic types of Monkeypox virus, the central African or Congo type and the West African type and the geographical division between the two types is Cameroon where both genetic types of the virus can be found. A systemic review of studies published till September 2020 has found that Monkeypox has till September 2020 appeared in10 African Countries and 4 non-African countries including Singapore, Israel, UK and USA [1]. Democratic Republic of Congo and Nigeria are the most affected countries.

The incidence of monkeypox has been reported to be as low as 0.64 per 100000 in 2001 to as high as 50 per 10000 in 2016 [1]. Case fatality ratio has been around 10%, with the central African type having a significantly higher case fatality ratio than the West African type. All the deaths due to Monkeypox had been reported to be inside Africa, and no fatal case has been reported yet outside Africa. There is quite a demographic shift in Monkeypox from deaths only among children less than 10 years of age to deaths in young adults with a mean age of 27 years after 2000 with no solid reason as yet [1].

Rodents are the most likely natural host of Monkey pox, though it has also been found in squirrels and Monkeys in Africa. Monkeypox has both zoonotic and human to human transmission. Zoonotic transmission occurs via direct contact with blood, body fluids or monkeypox lesions of infected animals. Inadequately cooked meat can also be contributing to it. Human to human transmission occurs via respiratory droplets, direct contact with skin lesions of those infected, or getting in contact with objects contaminated from an infected person [2]. Sexual transmission of Monkeypox or other members of orthopoxvirus has not been confirmed yet. We could only find some case reports in a literature about transmission of smallpox during sexual contact with the person who recently had been vaccinated against smallpox but all the reports suggested that it was not sexually transmitted in a true sense; rather it seemed to have transmitted due to a close contact during sexual encounter with skin lesion of the vaccinee, against Smallpox [[3], [4], [5], [6]]. Intrauterine transmission is also reported [7,8].

Clinical manifestation has been divided into invasion and skin manifestation periods. Invasion period is recognized by prodromal symptoms preceding skin eruptions, including fever, chills, headache, body aches, malaise, regional lymphadenopathy and sometimes vomiting. Cutaneous manifestations are observed after 1–3 days of appearance of fever and include successive stages of macules, papules, vesicles, pustules, crusting, and scars for 2–4 weeks. They start to appear first on head and neck region and then moves towards the periphery [9]. Clinical presentation of Monkeypox resemble that of Smallpox as both have non-pleomorphic skin eruptions.

Diagnostic tools include both serological testing and Polymerase Chain Reaction test. For PCR, it is important to get adequate sample from skin eruptions, stored in sterile tube and transported and stored in cold temperature. Serological testing cannot confirm monkeypox but can detect orthopoxviruses generally, as orthopoxviruses are cross reactive [10]. Though not widely available, European Medicines Agency (EMA) has recently authorized Tecovirimat for use against Monkeypox [11]. Intravenous Cidofovir and Brincidofovir can also be considered for its treatment, with brincidofovir being the safer option [12].

Small pox vaccine has been reported to provide 85% cross protection against Monkeypox [10]. With fading immunity among populations and non-availability of first generation small pox vaccine for the public, newer generation vaccines have been developed. ACAM2000 and MVA-BN (modified vaccinia Ankara-Bavarian Nordic) are two vaccines licensed for use in the USA. MVA-BN is specifically developed for Monkeypox and is live, non-replicating vaccine based on modified vaccinia Ankara, a live attenuated form of vaccinia virus. It is available in the US as Jynneos, in Europe under the brand name of Imvanex, and Imvamune in Canada [10,13].

Monkeypox outbreaks had been reported regularly in Africa before 2000. The first case of Monkeypox in humans outside Africa was reported in the USA, and pet prairie dogs were linked to it. These dogs were in contact with Gambian Pouched rats and Dormice imported from Ghana. That outbreak led to around 70 cases of Monkeypox in the USA, but no death, fortunately. Cases had been reported in other countries too including Israel, UK, and Singapore mainly after 2018 [10]. A large outbreak had occurred in Nigeria in 2017 in which around 500 people were affected with a case mortality ratio of 3%. Monkeypox seems to have recently spread to a number of countries including Spain, Canada, Australia, UAE [14]. Monkeypox cases have been reported in Spain recently and have been suggested to be originating from sexual contact at raves in Belgium and Spain [15]. News report cases of Monkeypox in Australia and Canada too [16,17]. U.K is the most severely hit country in 2022 and the cases have been increasing. As of now, around 900 cases have been reported in more than 25 countries (see Fig. 1).

**Fig. 1:**
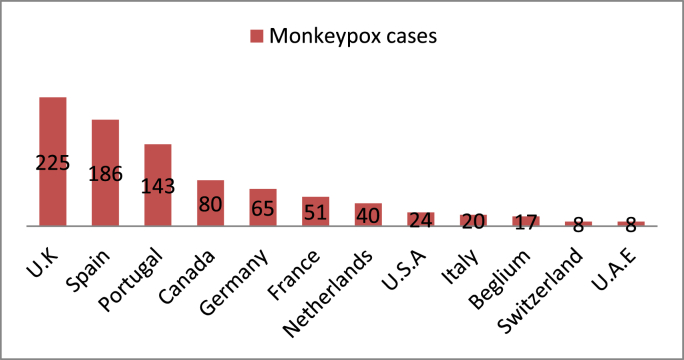
Number of cases of Monkeypox in each country in 2022 (as of 3rd June 2022). Data provided by Centers for Disease Control and Prevention (CDC), USA.

To reduce human-to-human transmission, it is important to avoid close contact with people infected with Monkeypox, including sexual contact. Health care workers are at most risk, and it is important to identify cases as early as possible. PPE should be made mandatory for health care workers dealing with such cases. Careful handling of samples is extremely important. Vaccines should be stockpiled for people at highest risk. Health screening and quarantine of individuals travelling from affected countries should be made mandatory. A quarantine period of 3 weeks has been suggested as of now.

## Ethical approval

Not applicable.

## Sources of funding

None to declare.

## Author contributions

*Mansoor Ahmed* conceived the idea.

*Haseena Naseer* and *Mansoor Ahmed* retrieved the data and draft the manuscript.

*Afnan Ahmad* and *Mateen Arshad* critically reviewed the draft and provided useful inputs.

## Trial register number

1.Name of the registry: Not applicable
2.Unique Identifying number or registration ID: Not applicable
3.Hyperlink to your specific registration (must be publicly accessible and will be checked): Not applicable

## Guarantor

Mansoor Ahmed.

Holy Family Hospital, Rawalpindi Medical University.

## Consent

Not applicable.

## Financial disclosure

None.

## Conflicts of interest

None to declare.
